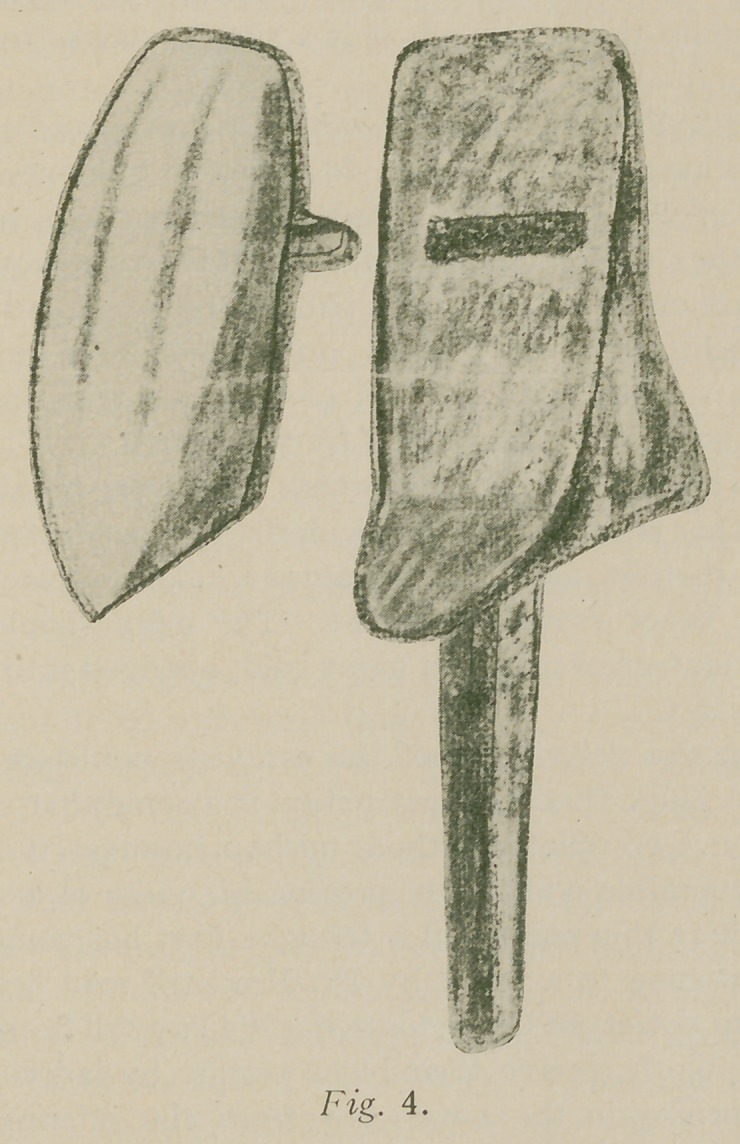# Best Methods of Avoiding Unnecessary Display of Gold in Bridge-Work

**Published:** 1908-01-15

**Authors:** W. A. Giffen


### BEST METHODS OF AVOIDING UNNECESSARY DISPLAY OF GOLD IN BRIDGE-WORK.

BY W. A. GIFFEN, D.D.S.

Read before the Detroit Dental Society, October 10, 1907.

Assuming that bridgework attached by crowns to the abutments is indicated and that the therapeutic or surgical treatment has been done that may be necessary to correct or arrest all pathological lesions which may affect the teeth or roots to be used as abutments, including pulp canals and surrounding tissues, the next step is to carefully fill the pulp canals and trim or shape the roots.

All remaining portions of the natural crowns should be excised on a level with the highest approximating gum festoon. The basal surface of the root should now be bevelled with a flat edge carborundum stone or root facer lingually and labially or buccally almost to the gum margin, with the exception that the lingual edge of the upper anterior teeth should be left longer than the labial in order to afford the greater mechanical resistance to the stress naturally imposed. The ledge of enamel remaining should be trimmed away and the periphery of the root tapered all around except at the labial or buccal portion which should simply be straightened. As much care should be exercised in doing this work as in the preparation of a compound cavity for an inlay.

An impression is now taken of the end of the root and a metal model made, a piece of forty gauge platinum large enough to make a hood for the root is then swaged over the metal model. The lingual and approximal portions of the collar being trimmed the desired length and the labial portion trimmed as short as possible, the cap is reswaged. The root canal should now be enlarged and the post fitted.

The post should be made of four-sided iridio-platinum wire of a gauge proportionate with the size of the root. It should be tapered at the point and flattened mesio-distally and should not be longer than the crown it is to anchor, I can see no necessity for using a longer post.

The canal should be reamed out just large enough to accommodate the post and so that the end will be tightly fitted into the canal. As a rule the directions of the canals should be followed in order to best preserve the strength of the root, except perhaps the upper anterior teeth when I think it best to enlarge the canals towards the lingual surface. In all cases, where roots to be used as abutments, are not parallel with each other it is necessary to enlarge the canals laterally to overcome the divergence or convergence as the case may be.

The labial portion of the basal surface of the root should now be shortened below the gingival line, the platinum hood adjusted by burnishing where necessary, punch small hole through hood into canal, push the post into place, remove and solder with platinum solder. In order to be sure that it fits right I replace on root and reburnish or swage, then invest and flow gold over the basal surface of the cap and collar to stiffen it, after which it should be filed and polished. The completed hoods are now placed on the abutments and an impression of the alveolar ridge taken with perfection impression material as hard as you can use it without hurting the patient. A model is made, and about the thickness of thin cardboard scraped off the ridge between the abutments. Take a piece of forty gauge platinum large enough to cover the ridge and long enough to reach the abutments, burnish or swage it to fit and reenforce it by soldering a strip of twenty-eight gauge platinum along the lingual edge of the saddle. Replace the hoods on abutments and place the saddle in place, and take another impression with perfection material stiff enough to force the saddle up tight against the ridge, coat the inside of hoods and saddle with whiting, invest and solder abutments and saddle together.

The completed foundation is now cleaned, enough gutta percha placed in the root canals to hold it, the posts warmed and placed in position. Impressions and models are again made and articulated on an anatomical articulator and we are ready to grind and fit the artificial teeth to the foundation. A strip of thirty gauge tin should be burnished and waxed to the saddle to allow for the thickness of gold used in making the hoods for the base of the crowns.

The cervical ends of the crowns should be ground to fit the saddle at the labial or buccal edges while the lingual portions should be shortened to allow for re-enforcement.

The periphery of the cervical end of each crown should be ground away around the lingual edge enough to allow the hood to cover it at least one eighth of an inch and as far labially as the post hole in the crown. Any of the ready-made porcelain crowns can be used in this way. Personally I like the White side crowns made for bridgework the best, for the reasons that the cervical end is made to conform to the general shape of the alveolar ridge being considerably longer at the labial edge. Again the post hole is closer to the labial portion allowing greater depth for the hole in the crown, also leaving a greater body of porcelain in the lingual portion to allow for more grinding so you can use a narrow saddle and still have room for whatever re-enforcement is necessary. I don't know any reason why this crown is not as good as any other, except perhaps, for the limited variety of shades.

After the crowns are fitted and the portions that have been ground are polished, posts of iridio-platinum should be made to fit holes in crowns. To make hoods for base of crowns invest on dental lac with cervical end exposed, place post in the crown, take piece of thirty-two gauge, twenty-four karat gold plate and punch a small hole in it, place over base of crown, forcing exposed end of post through hole in the gold and swage to place, remove and solder post and trim collar short as possible labially and as long as you can use it lingually, then reswage and burnish to place. The hood should fit snugly and still be easily withdrawn from the crown. The ends of the posts on basal surface of hoods should be ground off. Now strip off tin from saddle and abutments, place crowns in position waxing hoods to the foundation, remove the porcelains and complete the waxing process, and then invest and solder from the lingual side. In finishing the bridge, cut surplus platinum from the saddle close to the edge of the crowns on the labial side with knife-edged carborundum stones and then round off the metal edge and polish.

The saddle should extend lingually just enough to allow for sufficient re-enforcement and contour.

This style of bridge I consider practical and applicable for either upper or lower and for as many teeth as necessary provided you have abutments in the proper locations to carry it.

I use platinum for the saddle as it withstands the chemical action of the secretions so much better than gold as to rank first in compatibility with the tissues which takes most kindly to it, and while it fits closely to the tissues it does not press hard enough to cause a protracted lack of blood supply. The alveolar ridge is protected and very little if any absorption takes place under the saddle.

A bridge constructed in this way feels agreeable to the tongue and lips, besides it is the cleanest fixed bridge made, as no particles of dirt can get under it and even the cells exfoliated by the epithelium are taken care of by the tissues, and the exposed surfaces of the bridge can be kept clean as easily as natural teeth. The saddle also acts as a matrix to keep the solder where you want it exactly and allows you to utilize nearly all the space between the ridge and occluding teeth for porcelain so that the bite must be extremely short to prevent the use of this style of bridge.

Another point in favor of the saddle is that it acts as a stay to keep the abutment attachments in perfect position while soldering the case.

In using porcelain facings in place of crowns I prepare the abutments and hoods in the same way, use gold for backing the facings and grind the cervical end of facing to fit the labial edge of ridge, no saddle being required. I have used the Steele detachable facings occasionally for about two years and I must say they are proving very satisfactory. I have had a few of them crack off, however, I may have trimmed the backings a little too short. The most discouraging feature about the Steele facings is the limited variety of shades and moulds to select from.

The natural crowns of teeth should only be used as supports for bridgework when their condition and appearance is suitable. After filling pulp canals, cut cavity from pulp canal to surface of tooth approximating space to be bridged. The labial wall should be straight, the lingual and gingival walls flaring outward or away from labial and axial walls. Burnish platinum matrix to the outer cavity and then ream out pulp canal and fit iridio platinum post. Use a post long enough to bend in the shape of an L, so that when one part of post is in the canal the other will rest on floor of cavity and protrude from same far enough to reach hood or backing of the approximating dummy tooth of bridge. The matrix should now be placed in cavity, the post placed in position and soldered slightly to the matrix. Replace in tooth and reburnish margins, then invest and finish. This combination of post and inlay can be made for any tooth.

I like the staple crown for anchoring short bridges to teeth with healthy pulps better than any other form of partial cap. I make a plaster model of the lingual and approximal surfaces of the tooth and take a piece of forty gauge twenty-four karat gold plate large enough to cover model and punch four or five holes in the center of it and burnish over the model. I now prepare the tooth in the usual way using very thin platinum for the matrix. When staple and matrix are completed dry surface of tooth, smear a little vaseline over the surface, replace matrix and press a warm pellet of Peeso's wax over lingual surface of matrix. Now warm the gold hood which was swaged over metal model, place it over wax, gently press it to place, burnish margins which should overlap all joints. Remove from tooth, clean inner surface, coat it with whiting and invest including margin. Now burn out the wax and fill the space between the two layers of metal with solder through the hole in the lingual surface of the outer layer. This style of cap is very strong, which of course, is the most essential requirment. A hood or shoe made in this way and anchored to the lingual surface of teeth with small pins instead of the staple, can be used to good advantage in some cases if the work is well done.

Inlays or fillings do not make reliable attachments, except perhaps in molars and upper bicuspids, however, they are valuable when utilized as rests in the form of spurs or lugs on extended or continued and removable bridges.

The indications for removable bridgework summed up briefly are:

(1) In all cases where either fixed or removable bridgework is indicated, patients who will appreciate its artistic and hygienic properties sufficiently to take care of it.

(2) The loss of single bicuspids and molars and where the adjoining teeth are sound.

(3) Where the abutment teeth or roots are not in position to support fixed bridgework.

(4) Cases where there has been extensive resorption or loss of tissue.

The loss of a single molar forms a class of cases which I think is sadly neglected. The patient is apt to feel that the loss of one tooth is not of much importance, and many dentists treat the condition in the same way. If we would just stop and consider how many deformed jaws, and how many cases of mal-occlusion result from the loss of a single first molar or even a bicuspid in young patients, we should do better work.

I have bridged in a few single molars which are very satisfactory in the following way: Select suitable tooth to fill space, make groove on approximal surfaces of the teeth approximating the space to carry eighteen gauge iridio platinum wire. Now make hood for base of crown and short saddle for alveolar ridge and wax them together in proper positions. Cut the wire support long enough to reach from saddle through the groove and form a lug or spur to rest in the slot in the fillings or inlay of approximating teeth, and wax the ends to saddle, remove porcelain dummy tooth, invest and solder. When they are fitted properly they go to place with a click and are surprisingly solid. If the ridge is flat and you are afraid the dummy will rock, solder a clasp to the wire on distal surface long enough to clutch the mesio-buccal and mesio-lingual angles of the approximating molar.

Porcelain bridgework is an ideal type of prothesis when practicable. The two great reasons why it is not more universally used being—

(1) Porcelain is a vitrious and friable substance and as it has always been deemed necessary to fuse it to trusses and bars of various designs in order to make a bridge, thus destroying the integrity of the porcelain to the extent that it will not stand the stress it necessarily must undergo in the mouth, unless you can use a large bulk of porcelain.

(2) The difficulty of repair.

I firmly believe I have overcome both of these difficulties by the combination of a saddle, a matrix and cement. The only difference between a fixed or removable bridge being in the attachments. Prepare the foundation for the bridge as described in the first part of this paper, then re-enforce saddle on the lingual side of ridge with strips of iridio platinum to make it stiff, trim lingual edge and solder strip of clasp cold on edge against it and around lingual edge of hoods using a strip wide enough to leave the upper edge free, or so that it will form a ledge to finish against and protect the porcelain.

Select Davis crowns to suit the case, having some part of the labial portion of the base resting on the saddle. Make iridio platinum posts of round wire the same gauge as the ordinary Davis post and grind one end of post to fit the saddle, wax all posts in place and solder. Now take a piece of thin platinum large enough to cover entire saddle and abutments punch holes for posts a little larger than the posts, make tubes of inlay platinum large enough to fit over posts and solder in the holes in large piece of platinum, now place over saddle and burnish carefully all over saddle, when completely burnished, gently squeeze matrix tubes to posts, place crowns in position and remove from saddle and invest the under surface of the matrix with Pelton's investment and dry slowly but thoroughly, build procelain under and around edges of crowns, and biscut. Then add more porcelain and bake again, but it is well after the first bake to try on saddle to be sure the crowns are in place and to reburnish margins if necessary. When the baking has been completed strip off the matrix and it is ready to be cemented to saddle.

In cases where there has been extensive loss of tissue carry saddle as high labially or bucally as necessary and wire the edge the same as a continuous gum plate, make matrix in the same way. Build up and carve your porcelain to represent gums and finish or tint with gum enamel, of course, such a bridge should be made removable. In case of fracture, dissolve cement, make a matrix, replace broken parts on matrix and mend with new porcelain.

I have used this method in making a matrix to fit an individual Davis crown for about two years, or ever since Dr. Thompson demonstrated its use at one of our annual meetings. I got the idea of cementing a number of teeth in one block to a saddle from Dr. Kreit who uses it for making partial lower porcelain on a Watts metal base.

DISCUSSION.

Dr. G. C. Bowles: The title of Dr. Giffen's paper is significant as it implies that there has been and is an unnecessary display of gold in bridgework and that by modern methods such display may be eliminated. Minerva stepped full grown and perfect from the brow of Jove. Such things are possible with the gods, but the achievements of mortals usually have crude and imperfect beginnings.

When it was discovered that an ill-fitting partial plate might be replaced with a fixed and relatively permanent appliance, the accompanying disfigurment of gold abutments, gold cusps, gold tips, or a bridge entirely of gold was lightly regarded. Indeed it seems to have been hailed by many as a badge of distinction and gold was frequently called for by the patient where the dentist would have used porcelain, such became the desire to ape the well-to-do, who in the beginning alone could afford the gold bridge or crown. Then, too, it catered to that vicinity which finds expression in loud clothing, rings, bracelets, anklets, war paint, etc., according as the barbaric instinct is more or less refined. The modern "fine lady" who rode the white horse, while retaining the "rings on her fingers," exchanged the "bells on her toes" for a row of gold crowns just under her nose. Being easily made and yielding a good return to the operator the gold crown is still used indiscrimminately, often without the slightest regard to necessity, utility, comfort, permanance or appearance. The mouth of many a "fine lady" has been marred by the vulgar display of gold crowns in the anterior teeth and to add to the offense, the gums are frequently in an extreme degree of irritation—purple, swollen, congested, bleeding at a touch, due to the fact that the operator who will resort to gold crowns on the anterior teeth has no conception of the required root preparation, and is usually satisfied if the crown margins are only shoved up far enough to be out of sight. The glamor of gold in the mouths of all sorts of people, seen in the cars, on the streets, everywhere, all the time, and the fact that so much of it is far worse than merely unnecessary, makes Dr. Giffen's paper timely.

He outlines his technique for the preparation and construction of a saddle bridge—abutment crowns and dummies alike, being ready made porcelain crowns, cemented to place after the completion of the metal framework.

The bridge has several points of excellence. In the first place very little gold is exposed, it is strong, cleanly, artistic and the teeth not being subjected to the varying heat of the soldering process, are less liable to accident in the mouth. Should a tooth or all of the teeth break, they can be replaced without removing the bridge. This, in itself, is no small advantage.

A fixed saddle bridge, however, should be used only where absorption is complete, and usually only where from the shortness of the bite it is impossible to keep clean a bridge of the usual type. When of re-inforced construction the saddle is indispensable in some forms of porcelain bridges, adding the required rigidity and serving as a matrix for a considerable body of porcelain so necessary in this class of work. Accuracy in the construction and adjustment of the saddle bridge is imperative. Anything less will inevitably make of it a cesspool or a source of irritation.

I frequently make use of a bridge constructed in the same manner as described by the essayist, minus the saddle; that is, hoods like "A" Fig. 1, in the drawing are constructed for each crown and dummy, teeth placed in hoods (consolidated diatoric teeth are excellent for this purpose), assembled hoods waxed together, teeth removed, the metal part invested and soldered, and when finished, teeth cemented to place

"A" *Fig.* 1.

*Fig.* 2.

Such a bridge, with inlay and post for posterior abutment is shown in Fig. 2, has a wider range than the saddle bridge, and where indicated has all of its advantages.

Speaking of the root preparation the essayist says: "As much care should be exercised while doing this work as in the preparation of a compound cavity." I wish to emphasize this point. Prophylaxis is showing me more and more, that bands and ferrules of all kinds, extending up under the gums, are almost universally, a constant and aggravated source of irritation. It is possible to fit a band that will be non-irritating, but the process is so difficult that it is rarely ever done. I have eliminated the continuous band in my own practice almost entirely, using it only in such frail roots as threaten to split without it. It is not indicated in 5 per cent of cases. The simple disk on the bicuspids, the disk turned over on the lingual aspect to form a quarter band on the six anterior teeth is amply strong. The anterior roots should be trimmed to about a line below the gum line labially and from one-sixteenth to one-eighth of an inch above it lingually and the quarter band allowed to reach just under the free margin of the gum. Such a band is of the simplest construction, it is not irritating, does not encroach on the interproximal space and is practically as strong as the continuous band.

*Fig. 3.*

The essayist finds Steele's faceings useful, but their selection limited. I used them, for a time, with a like experience. I now employ the little scheme shown in the drawing. The facing is ground as desired and the pins bent to form a staple, "A" Fig. 3. Over the staple and to the facing is fitted a piece of twenty-four karat, thirty-six gauge gold forming a backing with a pocket for the pins. With flat nose pliers the outer margins of pocket are squeezed together close to pins, then cut through from the upper corner to base of pin, as shown at "B" Fig. 3. The upper half of this "wing" is then bent around against the pocket and the

*Fig.* 4.

lower half burnished to facing shown at "C",. The facing may now be removed and with gutta percha, or dental lac, or anything else suitable, fill the space under staple flush with pins, put back in backing and wrap the whole in tissue paper, put in swedger and swedge. Remove faceing, solder, after first filling pocket with whiting. Re-inforce backing with twenty-two karat thirty gauge plate, extending from tip to edge of pocket. Replace faceing, wax backing to cap or abutment, remove faceing, invest and solder. A crown with facing ready to be cemented to place is shown in Fig. 4.

I have used this method for over two years. In two cases the test is severe, yet I have not had one facing come off. Should they come off, it is a simple matter to replace them.

By grinding cutting edge of faceing thin, and having a rigid backing extending just to edge, no gold need show.

Concerning gold inlays, don't depend on them for abutments, they will inevitably fail. But if they are brought out to self-cleansing spaces, they will afford ample surface for the attachment of the dummy, and if anchorage into a root canal is secured with the aid of a square iridio-platinum post of good size as described by the essayist, "A" Fig. 2., then an abutment is secured which has a very great usefulness. This is applicable particularly to the bicuspids and molars where it may, with advantage, take the place of the gold or other crown abutment. The inlay should be of twenty-four or twenty-two karat gold, swedged into a platinum matrix.

From the description of the essayists porcelain bridge, I should judge that its construction is a somewhat exacting operation. Completed without mishap, however, it ought to be a serviceable and highly presentable piece of work.

I will at this point add a thought that has come to me since listening to a paper by Dr. Broomell read before the American Society of Orthodontists. We have all been taught that the teeth receive their blood supply by individual arteries opening in the lower jaw from the inferior dental artery, and the nerve supply along with it. Dr. Broomell has made some thousands of dissections and photographic slides of teeth, and in all stages of development up to the 12th year, and never in a single instance has he seen a single artery branching off from the main trunk; so it is evident, apparently, that the teeth are not supplied in that manner. The blood supply arises from the peridental membrane. If we irritate that peridental membrane, as we do frequently with gold bands, we are causing a condition of congestion at the source of the blood supply to the tooth. It is bad enough under the old plan of things where the tooth has an independent artery entering it at the apex, removed some distance from the source of irritation, but if the congestion is produced at the source of irritation and the blood supply cut off, we will have lack of nourishment and starvation of the parts. That accounts for so many of the teeth with bands around them, where the tooth is loosened in its socket—teeth that have been well crowned with gold and become, later on, too loose to be of any use.

Dr. Giffen. I have here a piece of bridgework which was given to me before the meeting opened by a member of the Society. Thinking that it might be interesting to you, the man for whom this bridge was made was drowned at sea in the year 1827. This bridge had been worn by him some years previous to his death, so it must be somewhere near 100 years old.

Dr. Ward. There are a few things in which my experience has differed somewhat from that of the essayist. One of the things is the use of pure gold or pure platinum for bands. I think that is one of our most serious mistakes—the use of pure material, gold or platinum, if we want to make a band that is to hold anything. Pure gold or pure platinum will hardly keep its shape while you fit it; it stretches too much. I find that iridio, platinum of 36 gage makes a nice band. For gold abutment stays, I have used coined gold for the bands almost exclusively. It has not the best color, and is hard to work, but when you get it placed it stays where you put it, and keeps its form. The principal objection is that of the working of coin gold or iridio-platinum because of its hardness. For the incisors I make the partial band, and also for many bicuspids, and I think it is fully as strong as a band, while it does away with the gold view entirely. All the forces being applied from lingual toward the labial, I think it is seldom that we need a band in these cases. However, if you are going to use a band, I think you should use a thin one, and something as hard as you can work.

One of the things I would speak of that has not been my experience, as it has the essayist's, is to place removable bridges upon poor roots, as I think a removable bridge should never be put upon poor root supports. Then, you ask, where *are* you going to put removable bridges? One of the most perplexing things we have to do is to tell where to put and where not to put the removable bridge. There are always two deciding factors to direct us: First, and above everything else, cleanliness; second, strength. If it is a case that is going to be battered to pieces and require attention right along, I would make it removable; if it cannot be kept clean, I should certainly make it removable.

My experience with porcelain bridges has been very limited, and particularly in connection with removable bridgework. A porcelain bridge cannot be made removable and be accurate; there is no solder joint we can make which will permit a porcelain bridge being made which will stand the fire. I have had no success with porcelain work, except in small cases and in the anterior part of the mouth.

The gold inlay as a bridge support must be made right. I do not think the gold inlay, in the ordinary sense in which it is used for filling, is much good as a bridge attachment, even if it is the best. It will not prevent the tooth from being split; it will not stand for a long span, but it may stand for a little while in a short span. The inlay that can be relied upon should extend well over into the occlusal surface of the tooth, as well as the approximal surfaces.

I want to speak of one or two cases I have done within the past six months. One: The replacing of the first bicuspid by gold inlay in the second bicuspid and gold filling in the distal side of the cuspid. The second bicuspid was a perfect tooth, and I had to cut the mesial and distal surfaces out to good sized areas and joined them across the occlusal. I had a compound inlay which covered mesial, distal and across the occlusal. The distal side of the cuspid was cut pretty freely away from the lingual, and a little nub on the end of a platinum bar set in cement; around that cement I packed a gold filling. I have no bands around either root, and I think it is sufficiently strong. The mistake made with the inlay is attaching it to a long case which is bound to shrink, and the inlay will not go into place, no allowance having been made for the shrinkage of the metals. The inlay should be attached to the bridge the last thing that is done, and your bridge should be constructed with the very smallest possible amount of solder put in. I think it is the coming attachment, especially in connection with the treatment of pyorrhoea and loose teeth.

The thing that I object to with a fixed piece of saddle bridge work is the difficulty that the patient has in keeping the saddle clean. If you have plenty of opportunity for the patient to get at it, and the saddle is not too concave, the patient may be able to keep it clean, but if it is too concave it is a bad thing.

Dr. Schaefer. Dr. Giffen's paper reminds me of an incident that occurred in my office a year ago. We were talking about bridgework, and Dr. Giffen was sitting in my chair resting. I think he was wondering how he could fill in a space that he has in the right side of his mouth. I said there ought not to be any trouble about that; simply cut off the cuspid and put on a Richmond and anchor with a gold crown back of that. He seemed to be very much surprised at a remark like that. He said:"Are you crazy? That cuspid is a live tooth." He evidently has changed his idea about cutting off a live tooth.

Dr. C. P. Wood. I have done lots of bridgework, some poor bridgework and some very good in some years that are gone, I believe that it is productive of the greatest good of anything we can do. Replacing one or more teeth in the mouth with bridgework, we can often do our patient a ood deal of good with bridgework, but it requires the finest discretion, as has been indicated here, to put in just the right thing. No two mouths ever present to any of us alike, so I say it takes the finest discretion to put in the right piece of work in every case. If we can use the proper judgment and select the case that is best adapted to that method and need, we are certainly going to do the patient a great deal of good.

A porcelain bridge is something that I have never had the audacity to tackle; that is, an all-porcelain bridge. I have seen several so terribly broken up and in such bad shape that I have really never dared to tackle it.

The bridge suggested by Dr. Giffen, a portion being removable, I think is a handy idea. A bridge attached to an inlay and a post, I believe is all right. I have several, especially a bicuspid attached to a molar in the lower jaw. Very often with the loss of the first molar we do not like to cut a sound tooth all down for a gold crown, as has been done in years past. I have anchored a post in such a tooth, and in some cases have been able to anchor a shallow post and an inlay in a live tooth, and have the case remain solid. I have taken out the pulp and anchored the post to a gold inlay, and the little shrinkage that takes place has not become a bar against that kind of bridge in my estimation, because with an inlay you can make it to overlap the edges and be left thin enough so that you can burnish and fit it very acceptably, and take care of all the shrinkage in a one-tooth bridge, and that is where it is employed usually, or for cases where you have only one tooth to put in, whether it is between two molars or a molar and cuspid. The one idea that I think we all have in mind now in bridgework is to avoid the band. If there is anything destructive of good, sound teeth and good, sound peridental membrane and gums, it is the band; and if any of you have tried to practice prophylaxis alongside of these bands, you have sworn good and bad that you would never put another band on if you could help it. It is utterly impossible to properly cleanse a root with a band such as we have been putting on for years back. I just rack my brains, all the time, to find some way of putting in a bridge without the band, and I think within a very short time no bands will be used unless they are made with perfectly flush joints; that is, the tooth ground in such a manner that the thickness of gold will just make a flush joint with the root. This can be done, and by so doing we have no irritation to the gum. I believe that more iridio-platinum and platinum should be used about the gum in bridgework. In fact, I think you must have all noticed in many mouths where porcelain and platinum crowns have been put on that many times they are not very well fitted, and yet I have noticed that these platinum bands did not collect the vicid secretions in the mouth as does the gold. I have been very much pleased with this discovery, and I have a mind to use more iridio-platinum and platinum bands than I have in the past on that account, because they do keep clean in the mouth, and from a prophylactic standpoint, they are much to be desired over gold.

Dr. J. J. Travis. I feel that the essayist has given us a great feast, and he has uncovered it a little too fast for me to assimilate it all. I also wish to congratulate Dr. Bowles on the manner in which he has discussed the paper. The arch from cuspid to cuspid would certainly produce a very aesthetic appearance made of porcelain, as suggested, and would in some cases be very good if it were a shorter bridge; and I should think it would answer the purpose very well, but not for men who cannot work in porcelain and do not have an opportunity and the facilities for making it. I understand from the paper that we are to avoid the needless display of the metals, if gold or platinum is used at all, and perhaps the essayist's method will solve the problem sufficiently.

I am using a bridge very similar to this in the abutments, but for the dummies between I use some form of porcelain crown, such as the Davis, but I prefer the S. S. White crowns. I cut out the mesial or distal and swage a cap and flow into it the solder, and in that way I get sufficient strength without that display of gold in the front. There is just a little gold that can be seen between the teeth at the base. If there are not more than one or two dummies between, I believe it is very serviceable and easier, and I have had no difficulty with them. This is not original with me, but I have been using it and find it to be very serviceable and it produces a very aesthetic appearance.

Dr. R. W. Bunting. There is one thing that has come to my mind in bridgework, and that is that I feel very loath to anchor any form of bridgework in teeth in such a way that if any part of it should break, I would be, as we say, decidedly up against it. I have seen so many bridges break; facings crack off and bridges break through an abutment, teeth have to be nearly ground to pieces in order to get these abutments out and put in order to replace a substitute. I wish somebody would devise some method or means whereby we can get these bridges out easily, or else to replace broken parts. That has been helped out by the Steel facings and the other forms that can be made over the ordinary teeth, and I am very much interested in this try-out of this new gutta percha preparation called Onilite, although I have tried it in a few cases, and they have not stayed with me. This, I understand, is a new preparation, something like gutta percha, which is used for setting various kinds of bridgework and crown work, by putting on a hot instrument, you can take the bridge out at any time. I set two bridges with it, and they were perfectly hard and solid, and in two days came back loose. I gave the bottle to Dr. Munnery, and he said he had half a dozen cases in which they remained in good shape. I should like to see some manner in which we can get off our bridges when they break, without mutilation of the teeth

DR. J. J. TRAVIS. In regard to this bridge that I made: if anything happens to any one of the crowns, it is an easy matter to fit another one on without taking the bridge out. In an all-porcelain bridge, if you break anything, it has all to come off, but with this one that I spoke of, you can replace any tooth if it is broken, just as easily as if it was a steel facing.

DR. HOFF. I do not know that I can add anything to the technical features of this bridge, because none of the bridges that have been presented here are bridges that I have made myself; that is, in exactly the form.

I have been quite a little interested in the form of bridgework designed by Dr. D. D. Smith in connection with his work in the treatment of pyorrhoea, and it seems to me that it is ideal. I have not made a removable bridge for many years, but I think for the removable bridge occasionally there may be possibilities, in the lower jaw particularly, where the removable bridges may be made, but they are so very few that I seldom have made them. The artistic features of Dr. Smith's bridgework appeals to me more strongly than any other that I know of. As you know he makes, not a saddle bridge exactly, but it is a bridge on a saddle principle; that is he makes each individual tooth a saddle. Each tooth is built up with the contour of the tooth which it replaces, of crown metal, which is made of platinum on one side and gold on the other; the gold is put outside and platinum inside. The idea is to have the metal very thin, so as to enable one to get the exact form contour, the lingual as well as the approximal surfaces of each individual tooth are made of metal, and the exposed view entirely of porcelain. This is set down directly on the gum in the form of a saddle, and assembled into a bridge of any dimensions. This gives the exact shape of the teeth and form of the arch, so that it presents the lingual surface of the normal tooth to the tongue, and it is of decided advantage in filling up the contour of the arch. Dr. Smith grinds the faces of these teeth a great deal, and re-shapes the whole tooth to make it conform to the adjoining natural teeth, and he gets the most artistic results I have ever seen. He does not in any case allow the occluding metal to run over the incised edges of the teeth, so that when you see his bridge in the mouth you cannot in many cases detect where the artificial tooth is and where the natural tooth is. There is no metal showing between the teeth. I have seen a number of his cases where he had very extensive bridges all around the arch, but from the outside no metal was exposed anywhere. Everyone says: "Why, such facings will break off easily, and when they do, you will have no means of repairing them except to remove the whole bridge." They do not break off readily, because he takes exceptional pains to secure proper occlusion; but when they do break off he repairs them so expertly without removing the bridge, that one is unable to find the repairs that have been made in the mouth. The insides of the dummies are made all hollow, and he fills them with cement; and that enables him to make these repairs without solder by grinding a long pin facing to fit and barbing its pins and re-setting it with cement. I showed one of these bridges in the clinic at the Detroit College two years ago. This form of bridge, I think, for artistic results, is the best that I know of. The only difficulty about it is that it is not as strong as some bridges should be. Dr. Smith uses, as attachments for his bridges, the natural adjoining teeth, many of which have been badly affected by pyorrhoea. He does not hesitate to destroy a pulp, and when he does he attaches his bridge by means of pins directly into the pulp chambers, making very secure attachments, often to cuspid or incisor teeth. A facing of metal to cover the lingual surface of an incisor tooth with the post running into the root canal is made, and in this way he gets an inconspicuous attachment to the tooth, and at the same time a very secure one. I have seen perhaps a dozen very extensive cases that had been worn for a number of years, and showed no indications of giving way, and were certainly much better than any porcelain or movable bridge could be for these cases, because they held the teeth firmly in place, and were perfectly artistic and serviceable. He was able to make just such occlusion as he wanted in every case, and the occlusion was as nearly perfect as is practicable to make occlusion in these cases. This bridge is ideal from a hygienic standpoint. The cement used here is not exposed to the secretions of the mouth in the same way that a cement filling would be, and does not dissolve away; it is kept in contact with the gum tissue, and because of this close contact there is always a little moisture under these dummies, which prevents the changing of its characteristics from alkali or abnormal to an acid solution, so that the cements do not dissolve out much, and no considerable deposits ever take place there. He passes floss silk under these bridges once a month, and instructs his patients how to do it themselves, so that they are kept clean. Dr. Smith told me that he has taken off some of these bridges that had been on for years, and he found no considerable amount of absorption of the cement.

Dr. Giffen. I am very happy to know that my crude paper was received in such a generous spirit.

In reply to the criticism of Dr. Bowles, advocating caps for bridge abutments, without bands, I would say that I like to have some grip on the periphery of the roots. I do not like deep bands, and in no case should they infringe on the soft tissues.

Dr. Ward's argument showing that an inlay and post in root canal is apt to split the tooth, I think is more applicable to a cap without a band. Anterior bridge abutments have to stand a great amount of lateral strain, and if you split one of these roots it is a terrible thing to happen. So if you can make the work stronger by using a band I think you ought to do so.

I never think of using a band for a single crown on a good root. I do not think it is necessary if you trim or shape the root, as explained by Dr. Ward.

In bridges I have described the frame work does not have to stand the heat at all after being completed, as it does not go through the porcelain oven, and so there is no chance to unsolder it.

The objection to the use of the saddle was not so strong as I expected, nearly every speaker admitting that in many cases they were all right. Like all other parts of the work, they must be properly adapted to the tissues, for they will fail.

Regarding Dr. Smith's system of bridgework, as explained by Dr. Hoff: It may be all right for Dr. Smith, or for those whose patients are trained as Dr. Smith says his patients are, but I cannot see many points in favor of it. It seems to me that it would mean a good deal of work to keep it clean on account of so many inter-approximal spaces.

Regarding what Dr. Schaefer said about the removal of live pulps, I would say that the individual idiosyncracies of our patients are some of the conditions we must overcome if we expect to obtain the best results in bridgework.

## Figures and Tables

**Fig. 1. f1:**
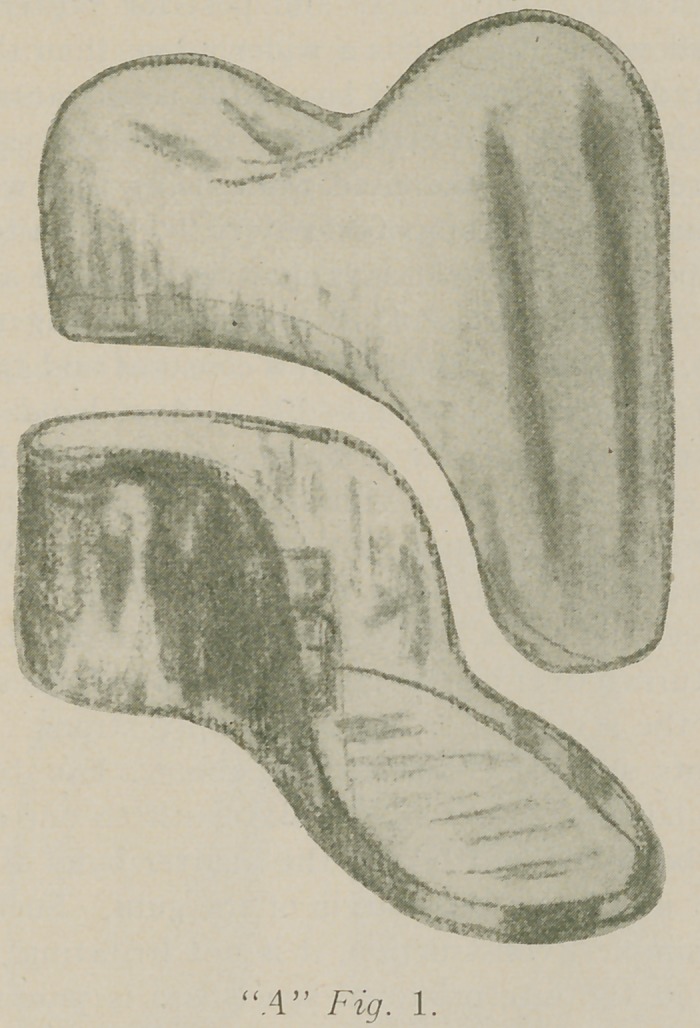


**Fig. 2. f2:**
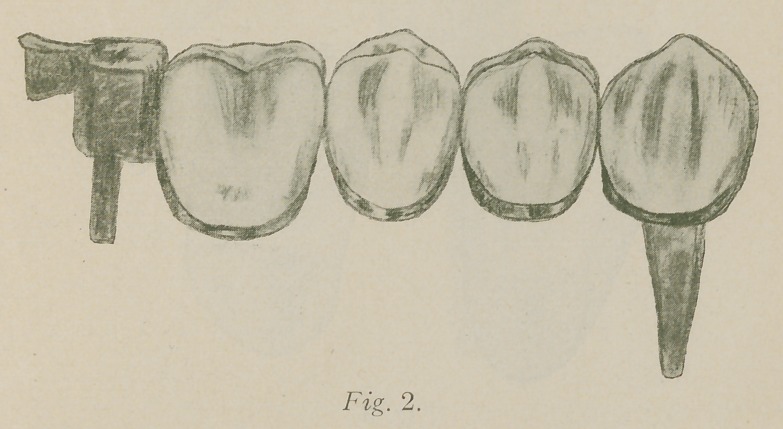


**Fig. 3. f3:**
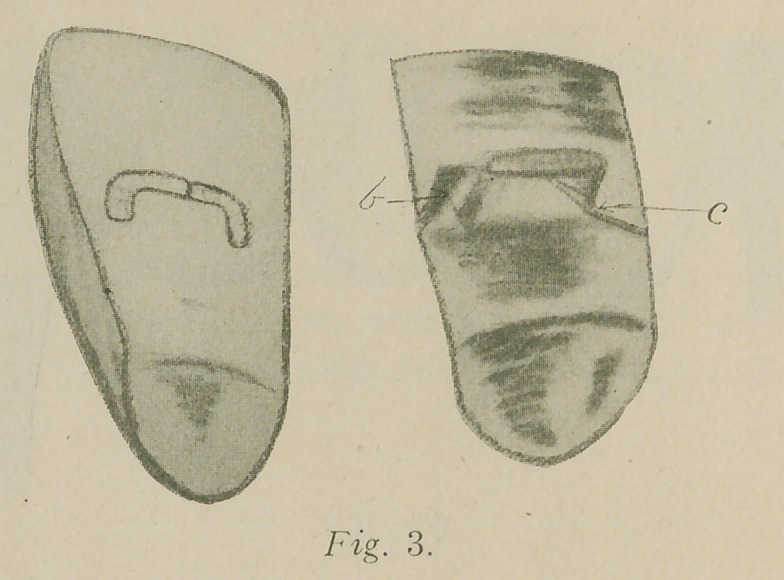


**Fig. 4. f4:**